# Ablation Therapy Combined with EGFR TKIs in the Treatment of Advanced Non-Small Cell Lung Cancer: A Meta-Analysis of Randomized Controlled Trials

**DOI:** 10.1155/2021/6624429

**Published:** 2021-05-07

**Authors:** Lu-Zhen Li, Jia-Ming Wu, Ting Chen, Liang-Chen Zhao, Juan-Na Zhuang, Hui-Si Hong, Ao Zhang, Hua-Tang Zhang, Can-Tu Fang

**Affiliations:** Zhongshan Affiliated Hospital, Guangzhou University of Chinese Medicine, Zhongshan 528400, China

## Abstract

**Objective:**

Systematically evaluate the efficacy of physical ablation combined with TKI in the treatment of advanced non-small cell lung cancer (NSCLC).

**Methods:**

We performed a comprehensive search of databases including OVID, PubMed, EMBASE, the Cochrane Library, and three Chinese databases (China National Knowledge Infrastructure, Wanfang Database, and Chongqing Weipu Database). The aim was to identify randomized controlled trials (RCT) investigating physical ablation as the treatment for advanced NSCLC. We also evaluated the methodological quality of the included studies and summarized the data extracted for meta-analysis with Review Manager 5.3.

**Results:**

A total of 9 studies, including 752 patients, were evaluable. The meta-analysis results show that the complete response rate (CRR) (RR: 2.23, 95% CI: 1. 46 to 3.40, *P* 0.01), partial response rate (PRR) (RR: −2.25, 95% CI: 1.41 to 3.59, *P* 0.01), and disease control rate (DCR) (RR: −2.80, 95% CI: 1.64 to 4.80, *P*< 0.01) of patients with advanced NSCLC who received physical ablation combined with TKI therapy were higher than those who did not receive physical ablation therapy. The control groups from seven of the studies had a total of 606 patients with targeted therapies and chemotherapy. The complete response rate was (CRR) (RR: 2.48, 2.4895% CI: 1.55 to 2.47, *P* 0.01), partial response rate (PRR) (RR: −1.66, 95% CI: 1.20 to 2.31, *P*< 0.01), and disease control rate (DCR) (RR: −2.68, 95% CI: 1.41 to 5.06, *P*< 0.01) for patients with advanced NSCLC who had received physical ablation combined with targeted therapies and chemotherapy, compared to patients who had not received physical ablation therapy. This difference was statistically significant. Above all, these results showed that the clinical efficacy of physical ablation combined EGFR-TKIs therapy (regardless of whether it was combined with chemotherapy) was better than that of EGFR-TKIs therapy alone.

**Conclusion:**

Physical ablation combined with TKI treatment in patients with advanced NSCLC can improve efficacy.

## 1. Background

Lung cancer is at the forefront of malignant tumors, in terms of both morbidity and mortality. Most lung cancer is diagnosed in its advanced stages such that most patients miss the opportune window for surgery. Instead, most receive multidisciplinary synthetic treatment. Combining systemic therapy with local therapy is always the focus of clinical inquiry. Since targeted treatment has become increasingly available in clinic, more and more patients with clear driver genes for non-small cell lung cancer have benefited from it. In this way, the appropriate use of molecular target drugs often achieves gratifying clinical results. However, with the passage of time, targeted drugs become resistant. Once this happens, drugs are rendered ineffective. Thus, methods for delaying resistance are worthy of scientific study. Physical ablation technology is a new technique for treating malignant tumors with physical technology. Common physical ablation techniques used in the clinic include radiofrequency ablation, microwave ablation, and frozen ablation [1–3]. The mechanism of action is similar in all forms of physical ablation. It entails inserting an electrode needle into the tumor tissue and producing a hot or cold effect based on the physical principle of denaturing tumor cell necrosis. In this way, it provides local treatment for malignant tumors. Several studies have demonstrated [4, 5] the clinical efficacy of ablation techniques. Although many articles have been published, both in China and abroad, on the combination of physical ablation and targeted therapy for advanced NSCLC, high quality systematic evaluation is lacking. For this reason, this study explores the efficacy of physical ablation joint targeted therapy for advanced NSCLC. A meta-analysis is conducted, with the aim of providing evidence in support of clinical work in choosing appropriate treatment options.

## 2. Methods

### 2.1. Search Methods

We performed a systematic search of the literature in OVID, PubMed, EMBASE, the Cochrane Library, and three Chinese databases (China National Knowledge Infrastructure, Wanfang Database, and Chongqing Weipu Database). The aim was to use the subject-word retrieval method to identify articles published from database inception through April 1, 2020. The retrieval model is as follows: *M* = (Lung Neoplasms or Lung Cancer or Non-Small-Cell Lung or Lung Carcinoma) and (Ablation, Radiofrequency or Radio Frequency Ablation or Ablation, Radio Frequency or Radio-Frequency Ablation or Ablation, Radio-Frequency or Cryosurgeries or Cryoablation or Cryoablations or Ablation Techniques or Microwave ablation) AND (EGFR tyrosine kinase inhibitor OR tyrosine kinase inhibitor) and (Randomized Controlled or Controlled Clinical or randomized or placebo or randomly or trial or groups or drug therapy).

### 2.2. Inclusion Criteria

(1) This study only includes randomized controlled clinical studies. There are no specific requirements for blinding methods; (2) pathological diagnosis of non-small cell lung cancer; (3) TNM phases III B to IV based onstage standards for primary lung cancer phase according to the AJCC eighth edition; and (4) age greater than 18 years old.

### 2.3. Treatment Means

(1) The treatment group was treated with physical ablation joint targeted therapy; physical ablation included radiofrequency ablation, microwave ablation, or frozen ablation; (2) control group was treated with the target treatment or combined chemotherapy, not limited to the specific target treatment drug; (3) the treatment of the control group was the same as that of the treatment group, except for the physical ablation therapy.

### 2.4. Outcome Indicators

(1) Main outcome indicators: complete response rate (CRR), partial response rate (PRR), and disease control rate (DCR) evaluated by solid tumor effect evaluation criteria (solid tumor response evaluation, RECIST1.1). (2) Secondary outcome indicators: life score quality, occurrence of common complications, PFS, and OS.

### 2.5. Document Screening and Data Extraction

Document screening and data extraction were performed by two researchers and were reviewed independently based on the inclusion criteria. The data extracted included the name of researcher, year of publication, number of cases, sex, and tumor staging. When there was questionable data, a third researcher would arbitrate.

### 2.6. Document Quality Evaluation

We conducted a quality evaluation of the included studies with Cochrane System Evaluation Manual 5.1. We assessed 7 aspects of randomized controlled studies: allocation of hiding, blinding methods, outcome evaluation blinding methods, incomplete outcome indicators, selective reporting, and other bias. According to the evaluation results, the included literature was divided into “high risk,” “low risk,” and “unknown risk.”

### 2.7. Statistical Methods

Meta-analysis was conducted with RevMan 5.3. Various methods were used for evaluation based on the data type. Two classification variables (e.g., CR rate, PR rate, and DCR) were analyzed with a relative risk (RR) and 95% CI as the effect analysis statistic. We used a fixed effects model for consolidation and analysis. The literature's heterogeneity was evaluated by a *Q* test and *I*^2^ statistical evaluation. If *I*^2^ ≤50% and *P* ≥ 0.10, the studies had better homogeneity. *I*^2^ >50% or *P* < 0.1 indicated that there was heterogeneity in the studies. Thus, we used a random effects model to analyze the factors that may have led to heterogeneity. This was assessed with sensitivity analysis and subgroup analysis. The published bias was evaluated by funnel plots, when there were over 10 research articles.

## 3. Results

### 3.1. Characteristics of Included Trials

**Figure 1:**
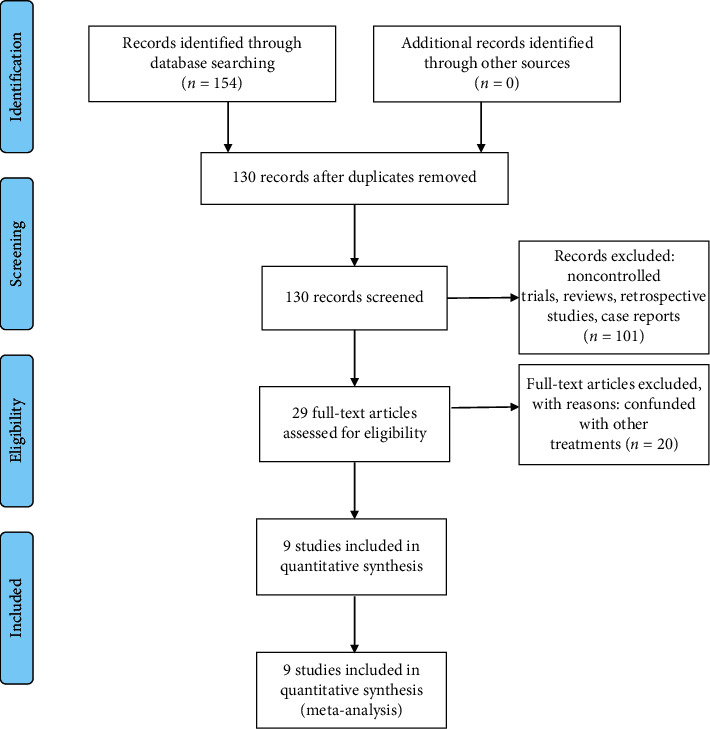
Literature retrieval and screening process.

Figure 1 is a flowchart of the literature retrieval. According to the search strategy, a total of 154 articles were retrieved, after excluding 24 duplicates, 38 reviews, 58 retrospective studies, and 5 case reports. An additional 20 articles were dropped after reading the full text and discovering their treatment had combined other local treatments such as radiotherapy or surgery. We were left with 9 randomized controlled clinical studies which were used in our analysis [6–14].

### 3.2. Data Extraction and Quality Assessment

**Figure 2:**
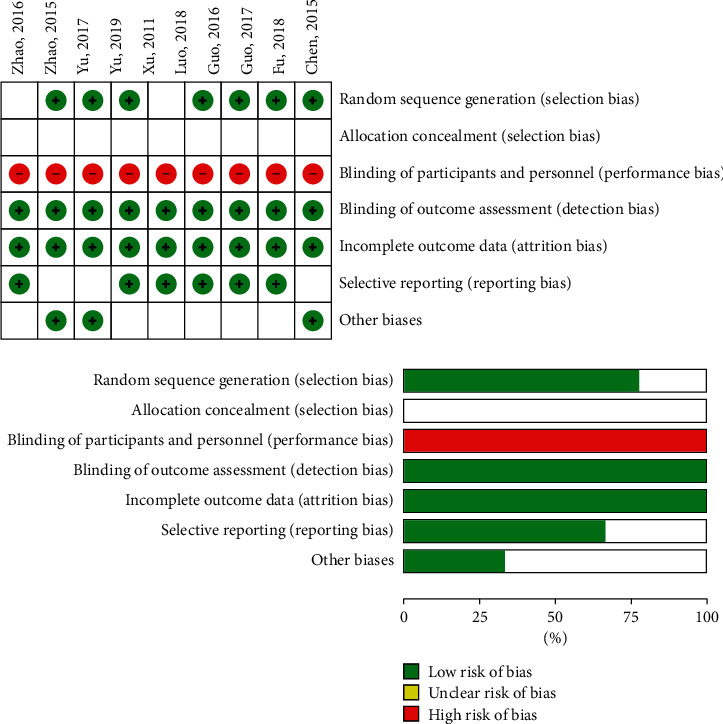
Risk of bias in included studies.

This study included 8 Chinese articles, 1 English article, and a total of 752 patients with advanced NSCLC. Among them, 376 patients had received physical ablation combined with targeted therapy or chemotherapy; the other 376 had received only targeted therapy or targeted therapy combined with chemotherapy. Of the 9 articles, 7 [7–9, 11–14] were about either image-guided percutaneous cluster electrode radiofrequency ablation (PRFA) or joint targeted therapy and chemotherapy, compared with targeted and chemotherapy treatment. 2 articles compared only microwave ablation joint targeted therapy and targeted therapy alone [6, 10]. One of the articles, in which the author's name was Zhang Wisdom, did not mention the randomization method. One paper [8] used a computer-aided random grouping method. The other 7 each used the random number table method to divide the experiment and control groups. Nine articles did not report a blinding method. One article reported the follow-up results, including overall survival (OS). Nine articles indicated that baseline data were comparable in both patient groups, but only 1 document [13] details the baseline data of the two patient groups. The basic characteristics of inclusion in the literature are shown in Table 1, and the quality evaluation is shown in Figure 2.

### 3.3. Analysis Results: CR Rates

**Figure 3:**
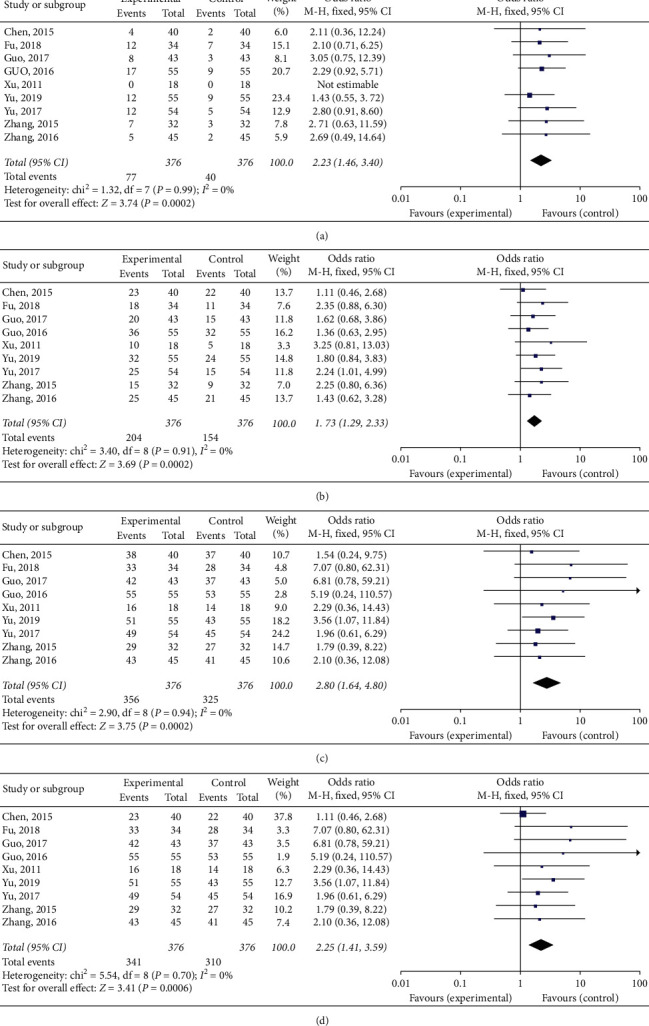
Meta-analysis results for clinical effect assessment. (a) CR forest plot, (b) PR forest plot, (c) disease control rate forest plot, and (d) ORR forest plot.

CR rates, PR rates, and DCR rates were reported in nine of the articles in this study. The results of the fixed effects model for the meta-analysis, as well as the forest plots, showed complete response rate (CRR) (RR = 2.23, 95% CI: 1. 46∼3.40, *P* < 0.01), partial response rate (PRR) (RR = 2.25, 95% CI: 1. 41∼3.59, *P* < 0.01), and disease control rate (DCR) (RR = 2.80, 95% CI: 1.64∼4.80, *P* < 0.01) among patients with advanced NSCLC who had received physical ablation combined with TKI therapy. These were all higher than for those patients who had not received physical ablation therapy. All of these findings were statistically significant. The control group had received targeted therapy combined with chemotherapy, in seven articles, with a total of 606 patients. Meta-analysis of the results for physical ablation combination therapy in patients with advanced NSCLC (compared to those accepting physical ablation) showed complete response rate (RR = 2.48, 95% CI: 1. 55∼2.47, *P* < 0.01), partial remission rate (RR = 1.66, 95% CI: 1. 20∼2.31, *P* < 0.01), disease control rates (RR = 2.68, 95% CI: 1.41∼5.06, *P* < 0.01), showing statistically significant difference, as shown in Figure 3.

### 3.4. Quality of Life (QOL)

Six articles reported on quality of life [7–9, 14], of which [11] 4 were based on the QLQ-C30 scale. One article adopted the quality of life score standard for tumor patients, including appetite, spirit, sleep, daily activities, and interpersonal communication. Patients' quality of life (QOL) was scored based on a total score of 25. Those with scores over 15 were rated as having good QOL. The proportion of patients with good QOL was compared between the two groups. One article [12] utilized a quality of life index questionnaire with six items—activities, daily life, health, total quality of life index, recent support, and overall spirit. Each item was rated either 0, 1, or 2. The scores were tabulated and categorized as follows: excellent (11 to 12 points), good (8 to 10 points), average (5 to 7 points), and poor (3 to 5 points). Those with <3 points were considered as having extremely poor quality of life. The latter two articles were inconsistent in their use of the scale. Therefore, we proposed combining the four articles [7–9, 14]. Finally, after reading the full texts of the 4 articles, it was demonstrated that study [8] only recorded five general functions and a total score for 3 symptoms in the quality of life scale. Individual scores for each item were not given. Thus, combination analysis was impossible. Study [7] recorded only one total score on the quality of life scale; the changes for each group and each score before and after treatment were unclear. Therefore, the second analysis could not be conducted. Two articles [9, 13] recorded the detailed score for five functions. However, the former recorded the specific value of the two groups of patients before and after treatment, while the latter only recorded the final score of the two patient groups. Thus, it was impossible to conduct a meta-analysis on the quality of life reports in the above-mentioned literature.

Although different recording scales were used, and the means of recording were inconsistent across studies, all of the studies showed that the quality of life scores for patients in the treatment group improved more than those of the control group after treatment.

### 3.5. Common Complications

**Figure 4:**
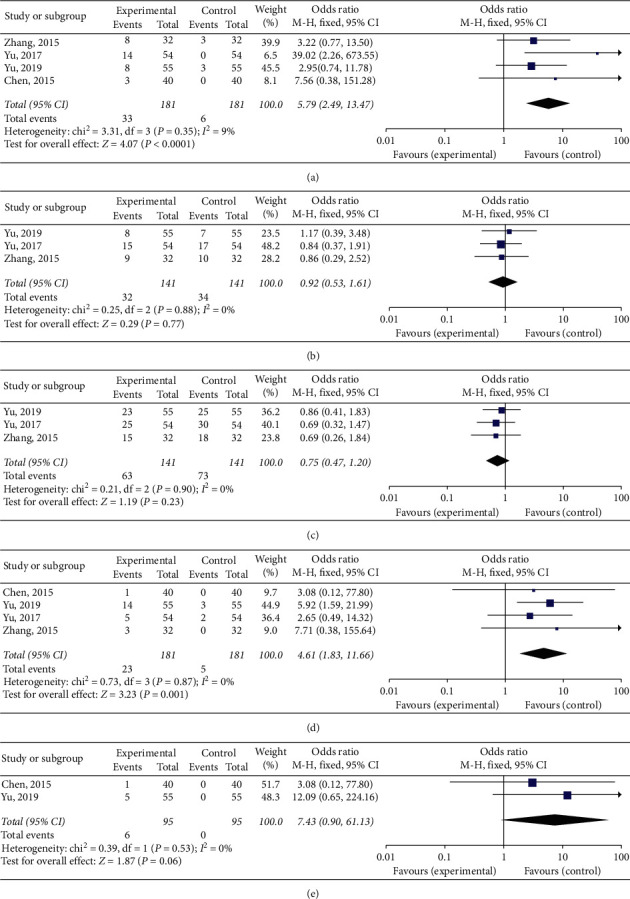
Meta-analysis of adverse reactions. (a) Forest plot of pain incidence; (b) forest plot diarrhea incidence; (c) forest plot of rash incidence; (d) forest plot of fever incidence; and (e) forest plot of pneumothorax incidence.

In this study, a total of 5 articles [6, 9, 11–13] reported adverse reactions, of which 4 [6, 11–13] reported the incidence of adverse reactions with pain. There was considerable homogeneity among them (*I*^2^, 9%, P, 0.35). The results of the fixed effects model showed that the incidence of pain in patients receiving physical ablation therapy combined with targeted therapy or chemotherapy was higher than that of patients receiving targeted therapy or chemotherapy alone. This difference was statistically significant (RR = 5.79, 95% CI: 2.49∼13.47, *P* < 0.01) (Figure 4(a)). Three articles [6, 11, 12] reported the incidence of diarrhea, with low homogeneity (*I*^2^ = 0%, *P*=0.88). This showed that, with a random effects model, there was no statistically significant difference between the incidence of diarrhea among patients that had received physical ablation combined with targeted therapy or chemotherapy and those that had received targeted therapy or chemotherapy alone (RR = 0.92, 95% CI: 0.53∼1.61, *P*=0.77) (Figure 4(b)). Rash incidence was recorded in 3 articles [6, 11, 12] with no statistically significant difference between the patients that had received physical ablation combined with targeted therapy or chemotherapy and those that had received targeted therapy or chemotherapy alone (RR = 0.75, 95% CI: 0.47–1.20, *P*=0.23) (Figure 4(c)). This was relatively homogenous (*I*^2^ = 0%, *P*=0.90). The 4 articles [6, 11–13] recorded incidence of fever and had low homogeneity (*I*^2^ −0%, P, 0.87) according to the fixed effects model. This showed that the incidence of fever in patients receiving physical ablation therapy combined with targeted therapy or chemotherapy exceeded that of patients receiving targeted therapy or chemotherapy alone. This difference was statistically significant (RR = 4.61, 95% CI: 1.83∼11.66, *P* < 0.01) (Figure 4(d)). Adverse pneumothorax reactions were also reported with low homogeneity (*I*^2^ = 0%, *P*=0.53). There were no significant differences in the incidence of pneumothorax in patients that had received physical ablation combined with targeted therapy or chemotherapy compared with those that received targeted therapy or chemotherapy alone (RR = 7.43, 95% CI: 0.90∼61.13, *P*=0.06) (Figure 4(e)). Yu Bo et al. [15] reported the incidence of liver damage and hemoptysis in patients that had received physical ablation combined with targeted therapy and chemotherapy. They found no statistically significant differences with patients that had received targeted therapy and chemotherapy alone (*P* > 0.05). Chen et al. [13] reported that the incidence of bone marrow inhibition in patients receiving physical ablation therapy combined with targeted therapy and chemotherapy was statistically significant (*P* > 0.05), compared with that of patients who had received targeted therapy and chemotherapy alone.

### 3.6. Long-Term Effects

Two articles [7, 13] reported [13] median PFS. One article reported that the median OS in patients with radiofrequency ablation and chemotherapy treatment was 16.4 × 1.54, better than the 15.6 × 1.32 in patients receiving targeted therapy and chemotherapy alone (*P* > 0.05). One article, by Xu Gu, reported a one-year survival rate. The 1-year survival rate of patients receiving radiofrequency ablation combined with targeted therapy was 12/18. This was higher than that of patients receiving targeted therapy alone (*P* < 0.05).

### 3.7. Published Bias Analysis

**Figure 5:**
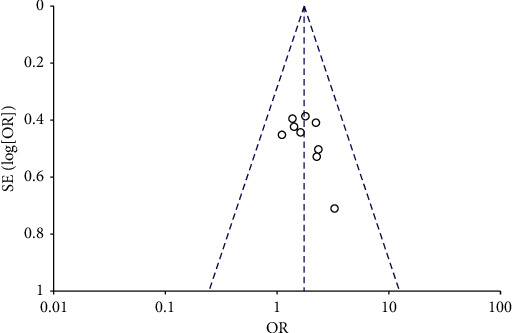
Bias analysis funnel chart.

Nine articles in this study were included in the funnel plot. Due to the asymmetry on both sides of the funnel plot, there may have been publication bias. Moreover, it is also possible that articles with negative results were not published (Figure 5).

## 4. Discussion

Of all malignant tumors, lung cancer has the highest incidence, causing immense financial costs at the population level. Many patients are already in advanced stages at the time of initial diagnosis and thus have missed the window for radical surgery. How to optimize the treatment strategy of advanced NSCLC and how to choose reasonable treatment at a specific time are the focus of clinical research. Since the IPASS study brought targeted therapy to clinical practice, patients with non-small cell lung cancer with clear driving genes have benefited. IPASS research has led to a series of studies that have established EGFR TKI as a first-line treatment for EGFR-positive patients. The median PFS for precision targeted therapy is 10–12 months, and many clinical studies have demonstrated the importance of targeted treatment in driving gene-positive non-small cell lung cancer [16–20]. Questions have arisen over what to do when resistance occurs. Appropriate targeted drugs can be identified for patients with the relevant drug resistance mechanisms, such as an ARUR3 study which brought ositinib to the clinic. This is the treatment for the first and second generations of TKI drug-resistant patients with the T790 M mutation. Data shows that about 60% of patients with the T90 M mutation can be administered targeted therapy with ositinib. However, due to various factors such as testing methods, drug availability, health policy, and other problems, far less than 60% of patients may actually be candidates for second-tier ositinib. Many patients have no drug for precise posttreatment drug resistance. There are various theories about this. One is that, based on clinical observations, approximately one-quarter of patients who discontinue TKI after progression experience explosive tumor growth. This is based on which TKI is recommended for continued use after progression.

Targeted therapy combined with chemotherapy has some advantages. However, several studies have demonstrated that this combination has limitations, and OS benefits are not obvious [16–20]. These results can be improved at this time by combining targeted therapy with local therapy [21]. Common physiotherapy ablation includes radiofrequency ablation therapy, microwave ablation therapy, and frozen ablation therapy. These are all forms of local physiotherapy; different physiotherapy methods have been demonstrated in a number of studies of the clinical efficacy of solid tumor therapy [22, 23].

In order to verify the role and safety of physical ablation therapy in patients with advanced NSCLC, this study conducted a rigorous and systematic meta-analysis. The results of this study show that patients who received physical ablation combined with TKI treatment have the following total remission rates, compared with patients who did not receive physical ablation therapy: CR: (RR 2.23, 95% CI: 1. 46 to 3.40, *P* 0.01), partial mitigation rate (PR) (RR −2.25, 95% CI: 1. 41 to 3.59 *P* 0.01), and disease control rate (DCR) (RR −2.80, 95% CI: 1.64 to 4.80, *P* 0.01). These were all statistically different. Seven of the studies' control groups had targeted therapy and chemotherapy, in a total of 606 patients. Meta-analysis showed that patients receiving physical ablation combination treatment for advanced NSCLC, compared with patients who did not receive physical ablation therapy, had full remission rate (RR 2.48, 2.4895% CI: 1. 55 to 2.47, *P* 0.01), partial mitigation rate (RR −1.66, 95% CI: 1. 20 to 2.31, *P* 0.01), and disease control rate (RR −2.68, 95% CI: 1.41 to 5.06, *P* 0.01). These were all statistically significant. These results show that the clinical efficacy of physical ablation combined with TKI targeted therapy (regardless of whether it is combined with chemotherapy) was better than that of simple targeted therapy or combination with chemotherapy. For long-term efficacy, the PFS of the physical ablation therapy combined with targeted therapy and chemotherapy was higher than that of the purely targeted therapy combined with chemotherapy (*P* < 0.01). However, the OS and 1-year survival rates were not available for meta quantitative analysis due to incomplete data in the literature. As for the quality of life, it was difficult to conduct meta-quantitative analysis because of the incomplete data on the scale and records of each aspect of the literature evaluation. However, each study showed that adding physical ablation therapy improved patients' quality of life scores. The meta-analysis results showed that the incidence of pain and fever in combination therapy was higher than that of pure targeted therapy and chemotherapy. Diarrhea, rash, and pneumothorax incidence made no significant difference in the incidence of common adverse reactions. Higher incidence of pain than that of the simple treatment group may have been due to minimally invasive operations. The high incidence of fever may have been related to the absorption heat caused by tumor necrosis after physical ablation, which is quicker and easier to treat.

## 5. Limitations

The principle of treatment for advanced non-small cell lung cancer can be comprehensive and based on systemic therapy. Physical ablation is characterized by minimal treatment, which has small surgical wounds and fewer effects on normal tissue, and requires less operating time than with surgery. As a minimally invasive local surgery, physical ablation can reduce the load of tumors in a short period of time. Additionally, it causes little delay in targeted therapy, chemotherapy, or other systemic treatments.

This analysis consisted of comprehensive literature retrieval and strict control of the inclusion standards. Regardless, there are still the following shortcomings: because this article is a secondary study of data from other literature, there are deficiencies: (1) only 1 corresponding English language study was retrieved, and all of the included studies were single-center. As a result, they need further confirmation. (2) The therapeutic effect's evaluation time after combination treatment varied in the included literature. This resulted in time bias. (3) The scale used to evaluate the quality of life was not uniform. Although most using the QLQ-C30 scale, there were variations in how each study recorded data. As a result, they could not be analyzed quantitatively. Also, the included literature reported less on long-term efficacy such as PFS and the OS of the combination of physical ablation and targeted therapy. This means that there is no evidence of physical ablation combination therapy's long-term efficacy. Therefore, the evaluation specification for the efficacy of physical ablation treatment for advanced NSCLC needs further exploration.

## 6. Conclusion

Physical ablation therapy has good near term clinical efficacy in patients with advanced NSCLC. Compared with purely targeted therapy or combined with chemotherapy, physical ablation combined therapy can improve patients' PR rates, DCR, and CR rates, assuming their safety is within the acceptable range. However, its long-term efficacy needs further assessment. New treatment options are available for patients with advanced NSCLC who cannot be treated with therapeutic surgery. However, there is still a need for large samples, and multicenter, high-quality randomized controlled trials, for further validation. Only then can it be included in the guidelines to benefit more patients with advanced non-small cell lung cancer.

## Figures and Tables

**Table 1:** General characteristics of included studies.

	Physical ablation joint targeting				Targeted or joint chemotherapy				Combined with chemotherapy	Outcome indicators
	Examples	Men	Women	Phases III-IV	Examples	Men	Women	Phases III-IV		
Guo et al., 2016	55	38	17	55	55	37	18	55	Yes, TP	①②③④⑦
Fu et al., 2018	34	24	10	34	34	23	11	34	Yes, TP	①②③④
Guo et al., 2017	43	31	12	43	43	29	14	43	Yes, TP	①②③④
Yu et al., 2019	55	26	29	55	55	28	27	55	No	①②③⑦
Xu et al., 2011	18	0	18	18	18	0	18	18	No	①②③⑤⑥
Zhang et al., 2015	32	Unknown	Unknown	32	32	Unknown	Unknown	32	TP, AP, or GP	①②③④⑦
U.S., 2017	54	Unknown	Unknown	54	54	Unknown	Unknown	54	TP, AP, or GP	①②③④⑦
Chen et al., 2015	40	21	19	40	40	20	20	40	Yes, TP	1 ②③⑥⑦⑧
Zhang, 2016	45	25	20	45	45	24	21	45	Yes, TP	①②③④

Outcome indicators: (1) full remission rate, (2) partial remission rate, (3) disease control rate, (4) quality of life, (5) 1-year survival rate, (6) no progression survival, (7) adverse reactions, and (8) OS.

## Data Availability

The data used to support the findings of this study are available from the corresponding author upon reasonable request.
